# Evaluation of Accessibility and Use of New Communication Technologies in Patients With Type 1 Diabetes Mellitus

**DOI:** 10.2196/jmir.4.3.e16

**Published:** 2002-12-20

**Authors:** Gabriel Giménez-Pérez, Maria Gallach, Edita Acera, Araceli Prieto, Olga Carro, Emilio Ortega, José-Miguel González-Clemente, Dídac Mauricio

**Affiliations:** ^1^Hospital de SabadellUnit of Diabetes, Endocrinology and NutritionInstitut Universitari Parc TaulíUniversitat Autònoma de BarcelonaSabadellSpain

**Keywords:** Diabetes mellitus, insulin-dependent, Internet, attitude to computers, patient education

## Abstract

**Background:**

The role of patients in the management and control of type 1 diabetes mellitus, a chronic disease, is well established. The advent of new communication technologies is expected to improve patients' access to health information. However, little is known about the extent to which patients with type 1 diabetes mellitus use the Internet to retrieve medical information and about the impact, if any, this retrieval has on their health status.

**Objective:**

To evaluate the accessibility and use of new communication technologies in a population of patients with type 1 diabetes mellitus.

**Methods:**

Patients with type 1 diabetes mellitus attending the Diabetes Clinic of the Hospital de Sabadell, Sabadell, Spain, in a 6-month period were asked to answer a structured questionnaire about education level, Internet accessibility, use of health-related Web sites, and mobile-phone ownership and use.

**Results:**

Of 302 patients with type 1 diabetes mellitus attending the Diabetes Clinic on a regular basis, 244 (115 men, 129 women) were interviewed (response rate 80.8%). Personal computers were owned by 58.2% of patients. Fifty-nine percent had access to the Internet, 39.3% had access to the Internet at home; however, only 36.5% were regular Internet users. Internet users were younger, more frequently men, and of higher education level. Among Internet users only 49.4% had ever accessed a health-related Web site. Internet users who had ever accessed a health-related Web site had a higher level of education, presented severe hypoglycemia more frequently, and were more likely to have access to the Internet at home. No differences were found in metabolic control between Internet users and nonusers or between Internet users who had ever accessed a health-related Web site and Internet users who had never accessed a health-related Web site. Of the 76.6% of the patients that owned a mobile phone, 96% used it more than once a week.

**Conclusions:**

The impact of new communication technologies might be jeopardized by the low rate of access and utilization of the Internet for health-related purposes. Because of their high rate of ownership and use, mobile phones show promise as a tool in health care communication technologies.

## Introduction

The increased use of the Internet by ordinary people is changing the way health care providers and the general population search for and retrieve medical information, which, in turn, modifies user-provider interaction and health care delivery. Studies have evaluated the use of the Internet in different medical conditions to assess its impact on patients' knowledge and well-being [1-4].

The role of patients in the management and control of type 1 diabetes mellitus, a chronic disease, is well established. Several studies have evaluated different Internet-based solutions for diabetes care [5-8]. However, we are not aware of any study assessing Internet accessibility and use among patients with type 1 diabetes mellitus. This data might be important for assessing the potential impact of Internet-based solutions for diabetes care in different settings. Therefore, we conducted this study to assess the accessibility and use of Internet resources in a population of patients with type 1 diabetes mellitus.

## Methods

Between October 2000 and March 2001 all patients with type 1 diabetes mellitus who attended the Diabetes Clinic of the Hospital de Sabadell, Sabadell, Spain, were asked to answer a structured questionnaire about education level, Internet accessibility and frequency of use, and access to health-related Web sites. Ownership and use of mobile phones was also evaluated. Demographic and clinical data regarding the level of metabolic control and the associated morbidity complications were retrieved from clinical records. Type 1 diabetes mellitus was diagnosed according to criteria published elsewhere [9].

**Statistical analysis**

Discrete and continuous variables were compared using the Pearson chi-square test and the Student t test respectively. Logistic regression analysis was performed using the stepwise method to predict use of the Internet and access to health-related Web sites. Differences between variables were considered significant when *P* value was less than .05. All analyses were performed using SPSS 11.0 software.

Internet accessibility was defined as the possibility of access to the Internet either at home, school, work, or other places. Internet users were defined as those accessing the Internet at least once a month. Access to health-related Web sites was defined as ever having accessed a health-related Web site. Mean HbA_1c_ (glycosylated hemoglobin) level was defined as the mean of all HbA_1c_ levels obtained during the 12 months before the interview. Presence of severe hypoglycemia was defined as any episodes of hypoglycemia requiring external help during the 12 months before the interview. Intensified diabetes treatment was defined according to usual criteria [10].

## Results

Of 302 patients with type 1 diabetes mellitus who attended the Diabetes Clinic at least once a year and who kept appointments for visits, 255 had an appointment during the study period. Of these, 11 failed to keep the appointment. Therefore, a total of 244 patients were interviewed. Patients not interviewed (n = 58) were older (41.5 [15.6] vs 34.3 [12.9] years; *P < .* 005), had a longer duration of diabetes (14.5 [11.7] vs 11.5 [9.1] years; *P < .* 05) and used intensified treatment protocols less frequently (60.3% vs 75%; *P < .* 05). Gender, presence of complications, and mean HbA_1c_ levels were not different among both groups. Clinical and educational data of interviewed patients are shown in Table 1.

**Table 1:** Clinical and educational characteristics of patients interviewed

	All patients interviewed* (n = 244)	Nonusers of Internet* (n = 155)	Users of Internet* (n= 89)	*P* value
Age (years)	34.3 +- 12.9	36.5 +- 14.1	30.6 +- 9.4	< .005
Gender (M/F)	115/129	65/90	50/39	< .05
Education level †				< .001§
University/high school	85 (35.4%)‡	36 (23.5%)‡	49 (56.3%)‡	
Secondary school	53 (22.1%)‡	37 (24.2%)‡	16 (18.4%)‡	
Primary school	102 (42.5%)‡	80 (52.3%)‡	22 (25.3%)‡	
Duration of diabetes (years)	11.5 (9.0)	12.8 (9.5)	9.2 (7.6)	< .005
HbA_1c_ (%)	7.60 +- 1.60	7.71 +- 1.58	7.41 +- 1.63	.160
Intensified treatment	181 (74.2%)	113 (72.9%)	68 (76.4%)	.547
Microvascular or macrovascular complications	86 (35.2%)	64 (41.3%)	22 (24.7%)	< .05
Severe hypoglycemia	22 (9.0%)	15 (9.7%)	7 (7.9%)	.634

^*^ Data are mean +- SD, mean (SD), n/n, or n (%)

^†^ Secondary school refers to the obligatory education between the ages of 12 and 16 in Spain; afterwards people can opt for high school which allows for University education (Spanish: Bachillerato Superior 16-18) or for other options

^‡^ Because there was no education data for 2 nonusers and for 2 users, these percentages were calculated based on the number of patients for which data was available

^§^ Grouping primary and secondary school

Of the 244 patients interviewed, 142 (58.2%) owned a personal computer, 144 (59%) had access to the Internet, 96 (39.3%) had access to the Internet at home; however, only 89 (36.5%) patients were Internet users. Of the 187 (76.6%) patients that owned a mobile phone, 180 (96.3%) used it more than once a week and 162 (86.6%) patients knew how to use the Short Messages System.

As seen in Table 1, compared with Internet nonusers, Internet users were younger, were more frequently men, were of higher education level, had diabetes of shorter duration, and had a lower degree of complications. In a logistic regression analysis after introducing all significant variables included in Table 1, only educational level, age, and gender predicted Internet use. Internet users owned personal computers (87.6% vs 41.4%; *P < .* 001) and mobile phones (87.6% vs 41.3%; *P < .* 001) more frequently than Internet nonusers.

Among Internet users only 44 (49.4%) had ever accessed a health-related Web site. Table 2 shows the characteristics of Internet users who had ever accessed a health-related Web site and Internet users who had never accessed a health-related Web site. As shown, Internet users who had ever accessed a health-related Web site had a higher level of education, presented severe hypoglycemia more frequently, were more likely to have access to the Internet at home, and were more likely to own personal computers. Level of education, severe hypoglycemia, and Internet access at home remained significant after logistic regression analysis of significant variables.

**Table 2:** Clinical and educational data of Internet-users

	Had ever accessed a health-related Web site* (n = 44)	Had never accessed a health-related Web site* (n = 45)	*P* value
Age (years)	30.5 +- 8.0	30.6 +- 10.6	.934
Gender (M/F)	22/22	28/17	.245
Education level†			< .05‡
University/high school	30 (68.2%)	20 (44.4%)	
Secondary school	8 (18.2%)	14 (31.1%)	
Primary school	6 (13.6%)	11 (24.4%)	
Internet at home	40 (90.9%)	23 (51.1%)	.001
Personal computer	42 (95.5%)	36 (80.0%)	< .05
Duration of diabetes (years)	9.4 (8.0)	9.0 (7.2)	.844
_1c_HbA1c (%)	7.171 +- 1.62	7.64 +- 1.62	.182
Intensified treatment	37 (84.0%)	31 (68.9%)	.091
Micro or macrovascular complications	11 (25.0%)	11 (24.4%)	.952
Severe hypoglycemia	7 (15.9%)	0 (0%)	< .01

^*^ Data are mean +/- SD, mean (SD), n/n, or n (%)

^†^ Secondary school refers to the obligatory education between 12 and 16 in Spain; afterwards people can opt for high school which allows for University education (Spanish: Bachillerato Superior 16-18) or for other options

^‡^ Grouping primary and secondary school

## Discussion

To our knowledge, this study is the first to evaluate the use of new communication technologies in patients with type 1 diabetes mellitus in the real world. Although the purpose of the study was to evaluate its use in a nonselected population, patients evaluated were significantly different from those not interviewed. Clinical characteristics of the latter group suggest that they may be less prone to use new technologies. Therefore, the present study may have overestimated the use of these technologies.

Internet utilization and demographic characteristics of Internet users compare well with national data about Internet use [11] and are lower than results obtained from other European countries with the exception of France and Ireland [12]. Internet users were of higher education level, younger, and predominantly men.

Patients looking for health information were, as expected, of higher education level and they were more likely to have access to the Internet at home. Additionally, they presented serious hypoglycemia more frequently. However, the low number of patients with this condition casts doubt on the significance of this result. No differences were found in metabolic control between Internet users and nonusers or between Internet users who had ever accessed a health-related Web site and Internet users who had never accessed a health-related Web site, contrary to other studies where Internet use has been associated with a better health profile [3].

The rate of use of the Internet for health purposes is in accordance with other studies [2,3]. Internet users who access health information on the Web are around 50% of all Internet users [1-4], a figure which can be considered low for chronic diseases in which patient's self-management is highly encouraged. Reasons for not accessing health information on the Web were not addressed in our study. Results from other studies suggest that lack of training in information technology is felt to be one of the main determinants for not retrieving medical information from the Web [1], an obstacle which can be easily overcome [13,14]. Other issues which can affect the patient's willingness to use the Internet as a health tool are related to the anxiety and stress derived from having different sources of information [2], lack of time [15], and poor readability [16]. Alternatively, quality of information, one of the major concerns of health professionals [16,17], although important for those who actually search for health information on the Web [15], does not seem to worry those who do not seek for it [1]. Another aspect to be considered is the lack of a specifically-designed, professionally-moderated Web page, which is felt by patients to be a reassuring tool [18] and might increase the rate of health-related Internet use. However, in the best case, this specific product would have been used by 59% of our patients with type 1 diabetes mellitus. Based on the profile of younger, male Internet users with a shorter duration of diabetes (Table 1), this percentage would have been lower in patients with type 2 diabetes mellitus, because (a) patients with type 2 diabetes mellitus are older (because type 2 diabetes mellitus usually starts in people older than 40 years) and (b) it seems that age is one of the determinants of Internet use.

The rate of ownership and use of mobile phones in our study is high. Use of wireless technology in health care has been evaluated mainly as a telemedicine tool [19,20] and patient-oriented tools are still under development [21]. We are not aware of any study exploring the role of Short Messages Systems as reminders or as empowerment tools. There is a need to explore the role of present and future mobile-phone technologies in health care delivery. However, these technologies may not yet be powerful enough to support the tools needed for delivery of health care.

In summary, although the advent of the Internet will probably change the way in which health care is delivered, at present its impact, according to our study, might be partially jeopardized by the rate of access to and utilization of the Internet for health-related purposes. Further studies are warranted to evaluate the needs and worries of patients to better address patient-oriented Internet-based solutions for type 1 diabetes mellitus. Because of their high rate of ownership and use, mobile phones show promise as a tool in health care communication technologies.

## Abbreviations

HbA_1c_: glycosylated hemoglobin
